# Naproxen affects osteogenesis of human mesenchymal stem cells via regulation of Indian hedgehog signaling molecules

**DOI:** 10.1186/ar4614

**Published:** 2014-07-17

**Authors:** Omar Salem, Hong Tian Wang, Abdulrahman M Alaseem, Ovidiu Ciobanu, Insaf Hadjab, Rahul Gawri, John Antoniou, Fackson Mwale

**Affiliations:** 1 Lady Davis Institute for Medical Research, Jewish General Hospital, McGill University, Montreal, Canada; 2 Division of Orthopaedic Surgery, McGill University, Montreal, Canada; 3 École Polytechnique de Montreal, Montreal, Canada; 4 Orthopaedic Research Laboratory, Montreal General Hospital, McGill University, Montreal, Canada; 5 Lunenfeld-Tanenbaum Research Institute at Mount Sinai Hospital, Toronto, Canada

## Abstract

**Introduction:**

We previously showed that type X collagen, a marker of late stage chondrocyte hypertrophy (associated with endochondral ossification), is constitutively expressed by mesenchymal stem cells (MSCs) from osteoarthritis patients and this may be related to Naproxen (Npx), a nonsteroidal anti-inflammatory drug used for therapy. Hedgehog (HH) signaling plays an important role during the development of bone. We tested the hypothesis that Npx affected osteogenic differentiation of human MSCs through the expression of Indian hedgehog (*IHH*), Patched-1 (*PTC1*) and GLI family members *GLI1*, *GLI2, GLI3 in vitro*.

**Methods:**

MSCs were cultured in osteogenic differentiation medium without (control) or with 0.5 μM Npx. The expression of collagen type X, alpha 1 (*COL10A1*), alkaline phosphatase (*ALP*), osteopontin (*OPN*), osteocalcin (*OC*), collagen type I, alpha 1 (*COL1A1*) was analyzed with real-time reverse transcription (RT) PCR, and the ALP activity was measured. The osteogenesis of MSCs was monitored by mineral staining and quantification with alizarin red S. To examine whether Npx affects osteogenic differentiation through HH signaling, the effect of Npx on the expression of *IHH*, *GLI1*, *GLI2*, *GLI3* and *PTC1* was analyzed with real-time RT PCR. The effect of cyclopamine (Cpn), a HH signaling inhibitor, on the expression of *COL10A1*, *ALP*, *OC* and *COL1A1* was also determined.

**Results:**

When MSCs were cultured in osteogenic differentiation medium, Npx supplementation led to a significant decrease in *ALP* gene expression as well as its activity, and had a tendency to decrease mineral deposition. It also decreased the expression of *COL1A1* significantly. In contrast, the gene expression of *COL10A1* and *OPN* were upregulated significantly by Npx. No significant effect was found on *OC* expression. The expression of *IHH*, *PTC1*, *GLI1,* and *GLI2* was increased by Npx, while no significant difference was observed on *GLI3* expression. Cpn reversed the effect of Npx on the expression of *COL10A1*, *ALP*, *OPN* and *COL1A1*.

**Conclusions:**

These results indicate that Npx can affect gene expression during osteogenic differentiation of MSCs, and downregulate mineral deposition in the extracellular matrix through IHH signaling. Therefore, Npx could affect MSC-mediated repair of subchondral bone in OA patients.

## Introduction

Symptomatic osteoarthritis (OA), characterized by cartilage deterioration and osteophyte formation, is the most common joint disorder, affecting primarily the knees, hips, and hands, and the predominant symptom is pain [1]. It is the major reason for seeking medical care and accounts for most of the use of non-steroidal anti-inflammatory drugs (NSAIDs) [2]. Clinical recommendations for the sympathetic treatment of OA include acetaminophen (Acet) and NSAIDs such as ibuprofen (Ibu), diclofenac (Dic), naproxen (Npx), and celecoxib (Cele) [3]. Unlike narcotics that target the central nervous system to alleviate pain, NSAIDs inhibit cyclooxygenase (COX) activity within the central nervous system and at the peripheral pain site to prevent the conversion of arachidonic acid into prostaglandins. Thus, NSAIDs can alter certain fundamental processes involved in the normal healing of injured tissues [4]. However, they are often inadequate and pain is the number one reason for undergoing joint replacement surgery [5].

Subchondral bone sclerosis and progressive cartilage degradation are widely considered as the hallmarks of OA. Despite the increase in bone volume fraction, subchondral bone is hypomineralized due to abnormal bone remodeling [6,7]. NSAIDs are used clinically to prevent ectopic bone formation that is also known as heterotopic ossification, and the efficacy of NSAIDs in the avoidance of heterotopic ossification has been documented in controlled clinical trials. Experimental studies also have documented the negative effects of NSAIDs on healing of skeletal tissues [8]. In earlier work, it was found that NSAIDs suppressed proliferation and induced cell death in cultured osteoblasts [9]. However, little is known about their effects on the subchondral bone repair in OA patients.

Mesenchymal stem cells (MSCs) are multipotent stromal cells capable of differentiating into multiple mesenchymal lineages, including osteogenic, chondrogenic, adipogenic, myogenic, and neurogenic lineages, under different conditions [10,11]. MSCs can differentiate to osteoblasts to form bone, while commitment of osteoprogenitor cells and differentiation into pre-osteoblasts, which eventually develop into mature osteoblasts, is a requisite [12]. To reverse or retard the degeneration of articular cartilage and repair the subchondral bone, MSCs can be employed in biological therapy for OA [13].

In the previous study, we found that NSAIDs can affect the expression of both hypertrophic and osteogenic genes in MSCs during expansion, and naproxen (Npx) showed a stronger effect on gene expression in MSCs than other drugs [14]. The effect of the drugs on gene expression in MSCs can influence the treatment of OA. Thus, it is important to understand whether Npx affects the osteogenesis of MSCs.

The Hedgehog (HH) protein family has been found in all vertebrates, since their original discovery in Drosophila. Indian hedgehog (IHH) regulates both chondrogenesis and endochondral bone formation [15,16]. It is known to stimulate bone formation via a positive feedback loop. Disruption of HH signaling results in severe skeletal abnormalities [17]. HH morphogens are secreted from the cells and bind to the receptor, Patched (PTC), to relinquish SMO from PTC suppression, thereby enabling activation of the GLI family of transcription factors, which are used as markers for HH signaling activity [18,19]. They are responsible for HH-induced lineage commitment during MSC differentiation [20,21].

In this research, the effect of Npx on hypertrophy and osteogenesis of MSCs was studied. Because IHH signaling can affect the osteogenesis of MSCs and other osteogenesis-related signaling pathways [21-23], the effect of Npx on IHH signaling was also studied to elucidate the mechanisms involved.

## Materials and methods

### Source and expansion of stem cells

Normal human MSCs were obtained from Lonza Walkersville Inc (Walkersville, MD, USA). According to the supplier, these cells were harvested from healthy human bone marrow and were positive for CD105, CD166, CD29 and CD44, but negative for CD14, CD34 and CD45. All cells were expanded in DMEM supplemented with 10% FBS, 100 U/mL penicillin, and 100 μg/mL streptomycin and were used within four passages. The culture medium and the supplement were from Wisent Inc (St-Bruno, QC, Canada).

### Cell culture

In every well of a six-well plate (Sarstedt, QC, Canada), 5 × 10^5^ MSCs were plated and cultured in expansion medium overnight. The floating cells were removed and the attached cells were cultured until the confluence was more than 90%. Then the cells were cultured in osteogenic differentiation medium for 3 days to allow the cells to adapt to the new environment. Afterwards, the cells were cultured in 3 mL/well of osteogenic differentiation medium with 0.5 μM Npx (Sigma-Aldrich, Oakville, ON, Canada). The cells cultured without Npx were used as control cells. To test the effect of cyclopamine (Cpn) on gene expression in MSCs, 0.5 μM cyclopamine (Sigma-Aldrich) was dissolved in the culture medium. The osteogenic differentiation medium was prepared with high-glucose DMEM containing 10% FBS, 0.1 μM dexamethasone, 10 mM β-glycerophosphate, 50 μM L-ascorbic acid, 100 units/mL penicillin, and 100 μg/mL streptomycin.

### Total RNA isolation

After MSCs were cultured for 3, 6 and 12 days, they were washed with PBS and total RNA was extracted using Trizol reagent (Invitrogen, Burlington, ON, Canada) according to the protocol from the supplier [11,14].

### Reverse transcription and real-time PCR

First, 1 μg total RNA isolated from the cells was digested with DNase I (Invitrogen) according to the protocol of the supporter. Then, the purified RNA was reverse-transcribed as described previously [11,14]. Briefly, 1 μg RNA was mixed with random primers (final concentration 0.15 μg/μL), dNTP mixture (final concentration 0.5 mM), and DEPC-treated distilled water with a total volume of 12 μL. After the solution was incubated at 65°C for 5 minutes, it was mixed with a first-strand buffer, Dithiothreitol, RNaseOUT, and Superscript II reverse transcriptase with a final volume of 20 μL. Then, the solution was incubated at 45°C for 50 minutes to perform the reverse transcription and then at 70°C for 15 minutes to inactivate the reverse transcriptase. For LightCycler real-time PCR, a master mix of the following reaction components was prepared with the final concentrations at 10 μL SYBER PCR master mix (Qiagen, Mississauga, ON, Canada), 8 μL distilled water, 0.5 μL forward primer (final concentration 0.25 μM), and 0.5 μL reverse primer (final concentration 0.25 μM). To each 19 μL master mix, 1 μL of cDNA was mixed as a PCR template. The sequences of primers are in Table 1. The reaction conditions included one cycle of PCR initial activation step (95°C for 15 minutes, 20°C/s ramp rate), 45 cycles of amplification and quantification (94°C for 15 s, 57°C for 30 s, 72°C for 30 s), one cycle of melting curve (65°C to 95°C with heating rate of 0.1°C/s with a continuous fluorescence measurement), and a final cooling step to 4°C.

**Table 1:** Primer sequences

**Gene**	**Sequence**	**Size (bp)**
*ALP*	Forward (1397–1416): CCACGTCTTCACATTTGGTG	196
	Reverse (1573–1592): AGACTGCGCCTGGTAGTTGT	
*COL1A1*	Forward (3982–4001): GAGAGCATGACCGATGGATT	178
	Reverse (4140–4159): CCTTCTTGAGGTTGCCAGTC	
*COL10A1*	Forward (1670–1690): AATGCCTGTGTCTGCTTTTAC	130
	Reverse (1779–1799): ACAAGTAAAGATTCCAGTCCT	
*GAPDH*	Forward (113–133): TGAAGGTCGGAGTCAACGGAT	181
	Reverse (273–293): TTCTCAGCCTTGACGGTGCCA	
*GLI1*	Forward (676–695): AAGCGTGAGCCTGAATCTGT	189
	Reverse (845–864): CAGCATGTACTGGGCTTTGA	
*GLI2*	Forward (199–218): CGACACCAGGAAGGAAGGTA	203
	Reverse (382–401): TGCACAGAACGGAGGTAGTG	
*GLI3*	Forward (2285–2304): CTTTGCAAGCCAGGAGAAAC	163
	Reverse (2428–2447): TTGTTGGACTGTGTGCCATT	
*IHH*	Forward (519–538): CGGCTTTGACTGGGTGTATT	219
	Reverse (718–737): AAAATGAGCACATCGCTGAA	
*OC*	Forward (20–39): TGAGAGCCCTCACACTCCTC	151
	Reverse (170–151): CGCCTGGGTCTCTTCACTAC	
*OPN*	Forward (759–778): TGAAACGAGTCAGCTGGATG	162
	Reverse (920–901): TGAAATTCATGGCTGTGGAA	
*PTC1*	Forward (1460–1479): TCAGCAATGTCACAGCCTTC	248
	Reverse (1688–1707): GTCGTGTGTGTCGGTGTAGG	

The crossing points (CPs) were determined by the Light-Cycler software 3.3 (Roche Diagnostics, Indianapolis, IN, USA) and were measured at constant fluorescence level. The ratio of gene expression relative to *GAPDH* as the reference gene was determined by the following equation [11,14]:

$$\mathrm{Relative}\mathrm{ratio}=\frac{2^{\mathrm{\Delta C}P_{\mathrm{target}}\left(\mathrm{control}-\mathrm{sample}\right)}}{2^{\mathrm{\Delta C}P_{\mathrm{reference}}\left(\mathrm{control}-\mathrm{sample}\right)}}$$

### Alkaline phosphatase activity

After MSCs were cultured in osteogenic differentiation medium with or without Npx for 3, 6, and 12 days, the cells were lysed and ALP activity was assayed with StemTAG™ Alkaline Phosphatase Activity Assay Kit (Colorimetric) (Cell Biolabs Inc, San Diego, CA, USA) according to the protocol from the supplier.

### Mineralization analysis with alizarin red S

Osteogenic differentiation was monitored by mineral deposition with alizarin red S staining as previously described [11]. To quantify the matrix mineralization, alizarin red S was extracted with 1 mL/well 100 mM cetylpyridinium chloride (Sigma-Aldrich) and measured at 570 nm.

### Statistical analysis

Statistical analysis was performed using one-way analysis of variance (ANOVA), followed by Fisher’s protected least significant difference post hoc test, using Statview (SAS Institute, Inc). The results of three experiments with MSCs from three different donors were assessed, and the values were reported as mean ± SD. The significance was defined as a *P-value* <0.05.

## Results

**Figure 1:**
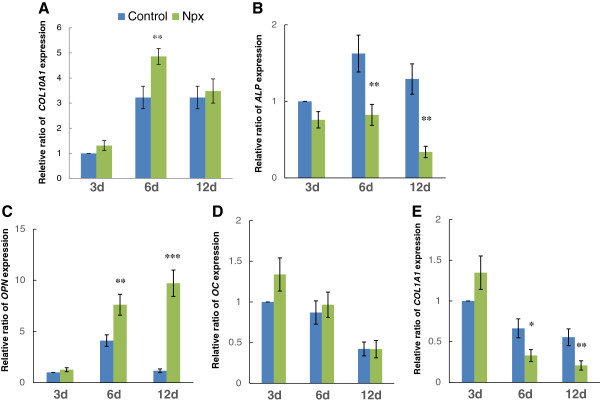
**Relative ratio of *COL10A1 *(A), *ALP *(B), *OPN *(C), *OC *(D) and *COL1A1 *(E) gene expression in mesenchymal stem cells (MSCs) cultured in osteogenic differentiation medium without (control) or with 0.5 μM naproxen (Npx).** The expression of *COL10A1* was detected after the cells were cultured for 3, 6 and 12 days. The results are shown as mean ± SD of three independent experiments with MSCs isolated from three different donors. *P <0.05 versus control; **P <0.01 versus control; ***P <0.001 versus control.

Message expression for various matrix protein genes was analyzed after culturing MSCs in osteogenic conditions supplemented with or without Npx for 3, 6 and 12 days. *COL10A1* is a marker gene for hypertrophic chondrocyte differentiation [24,25]. At day 6, the *COL10A1* message was increased significantly with Npx supplementation in the culture medium (*P* = 0.007), while no significant difference was found at days 3 and 12 (Figure 1A). Alkaline phosphatase (*ALP*), osteopontin (*OPN*), osteocalcin (*OC*) and *COL1A1* are important genes that define the osteogenesis of MSCs [24-26]. To test the effect of Npx on osteogenesis of MSCs, the expression of these genes was examined after MSCs were cultured in osteogenic differentiation medium with or without Npx for 3, 6 and 12 days. When MSCs were cultured with Npx for 6 and 12 days, message levels of *ALP* were decreased significantly compared with control (*P* = 0.007 on day 6; *P* = 0.001 on day 12), while no significant difference was observed on day 3 (Figure 1B). Npx increased *OPN* expression significantly on day 6 (*P* = 0.006) and day 12 (*P <*0.001) respectively, while no significant difference was observed on day 3 (Figure 1C). Treatment with Npx did not result in a significant change in *OC* expression (Figure 1D). Npx decreased the expression of *COL1A1* significantly on day 6 (*P* = 0.013) and day 12 (*P* = 0.007), while no significant effect was observed on day 3 (Figure 1E).

**Figure 2:**
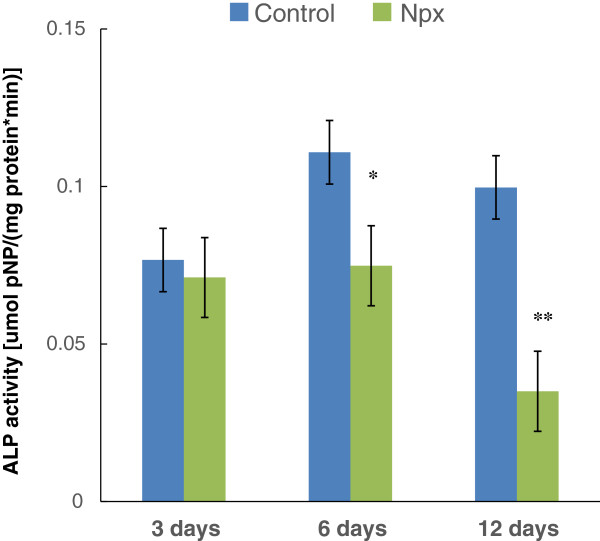
**The activity of alkaline phosphatase (ALP) in mesenchymal stem cells (MSCs) cultured in osteogenic differentiation medium without (control) or with 0.5 μM naproxen (Npx) for 3, 6 and 12 days.** The results are shown as mean ± SD of three independent experiments with MSCs isolated from three different donors. *P <0.05 versus control; **P <0.01 versus control.

**Figure 3:**
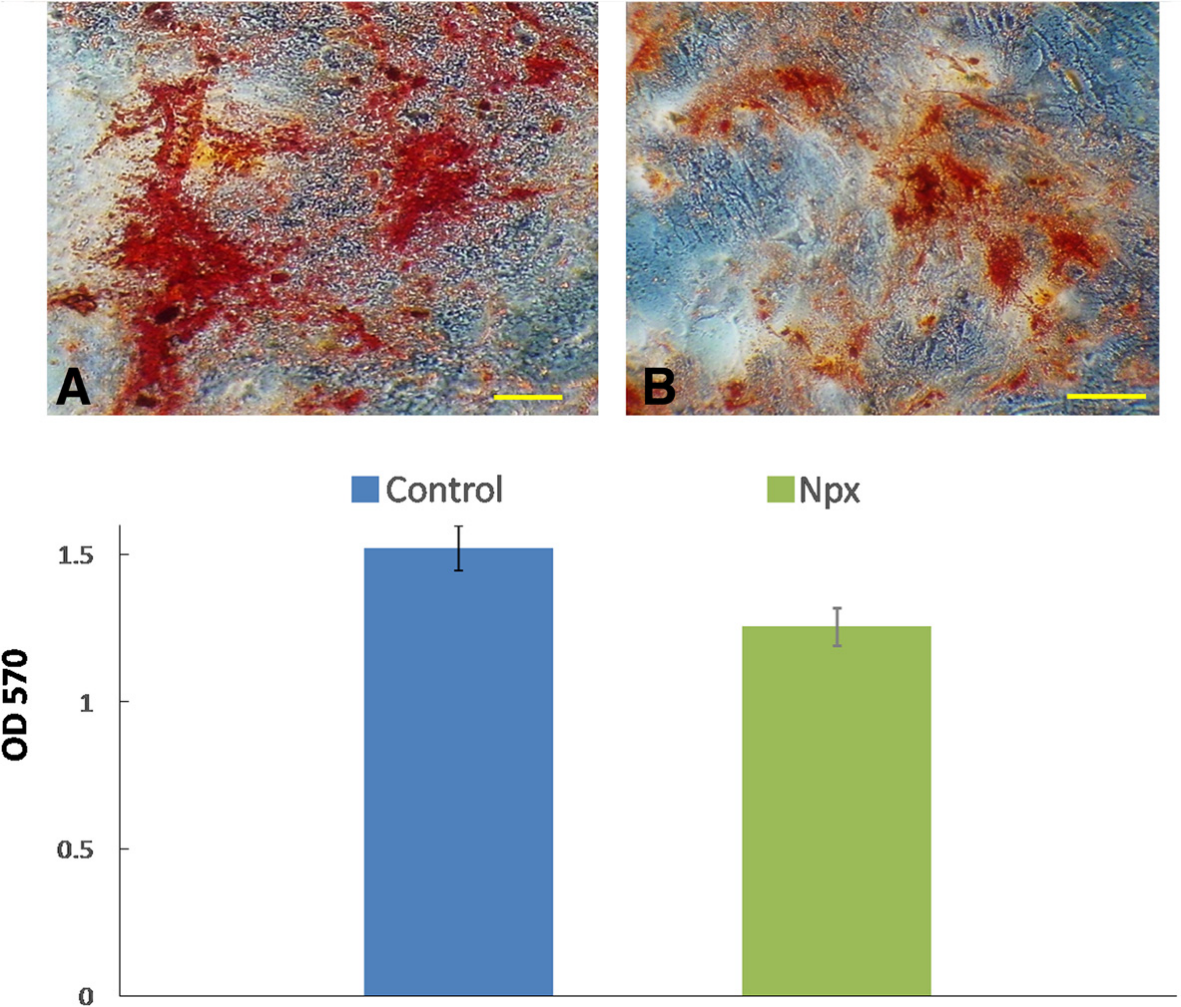
**Effect of naproxen (Npx) on mineral deposition by mesenchymal stem cells (MSCs) cultured in osteogenic differentiation medium.** Alizarin red S staining of mineral deposition in the extracellular matrix after MSCs were cultured in osteogenic differentiation medium without (control) **(A)** or with 100 μg/mL Npx **(B)** for 21 days (bar represents 50 μm); **(C)** the absorbance value of solubilized alizarin red S measured at 570 nm. The results are shown as mean ± SD of three independent experiments with MSCs isolated from three different donors. *P* <0.05 was determined as statistically significant.

As Npx suppressed *ALP* gene expression (Figure 1B), we next tested the effect of Npx on ALP activity (Figure 2). Npx significantly decreased ALP activity when MSCs were cultured with Npx for 6 days (*P = 0.03*) and 12 days (*P* = 0.004), but did not significantly decrease ALP activity on day 3 (Figure 2).In order to verify that MSC cultured in osteogenic differentiation medium with Npx for 21 days results in the deposition of mineral, they were visualized by staining the cells with alizarin red S, which detects calcium deposition. Red staining was present throughout the cultures (Figures 3A,B). With Npx, there appeared to be a decrease in the extent of matrix mineralization. When alizarin red S was extracted from the matrix, mineral deposition in the presence of Npx decreased although not significantly compared with control cells (Figure 3C).

**Figure 4:**
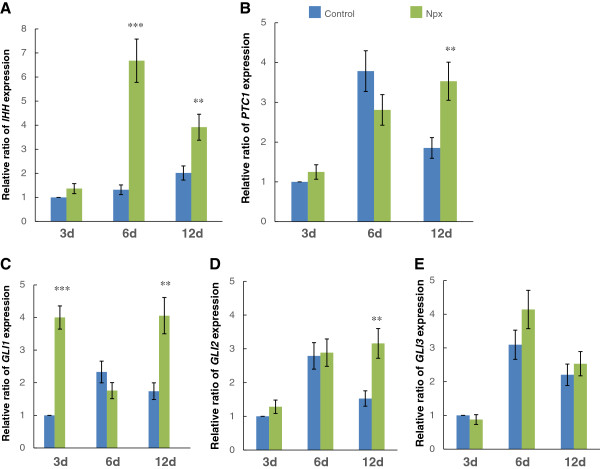
**The relative ratio of *IHH *(A), *PTC1 *(B), *GLI1 *(C), *GLI2 *(D) and *GLI3 *(E) expression in mesenchymal stem cells (MSCs) cultured in osteogenic differentiation medium without (control) or with 0.5 μM naproxen (Npx) for 3, 6, and 12 days.** The results are shown as mean ± SD of three independent experiments with MSCs from three different donors. **P <0.01 versus control; ***P <0.001 versus control.

IHH signaling is an important pathway in osteogenic differentiation, and is defined by *IHH*, *GLI1*, *GLI2*, *GLI3* and *PTC1* genes [18,19]. The effect of Npx on the expression of these genes was therefore assessed. When the cells were cultured in osteogenic differentiation medium with Npx, the expression of *IHH* increased significantly on day 6 (*P* <0.001) and day 12 (*P* = 0.006) compared with that in control cells (Figure 4A). This difference was not observed on day 3. The expression of *PTC1* (*P* = 0.006), *GLI2* (*P* = 0.005) increased significantly on day 12 compared with that in control cells (Figure 4B,D), while the expression of *GLI1* increased significantly on both day 3 (*P* = 0.001) and day 12 (*P* = 0.003) (Figure 4C). No significant effect of Npx on the expression of *GLI3* was observed (Figure 4D).

**Figure 5:**
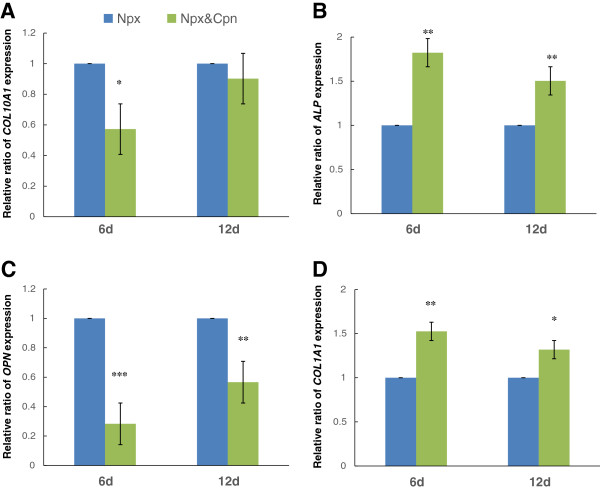
**The effect of the Hedgehog (HH) signaling pathway inhibitor cyclopamine (Cpn) on the expression of *COL10A1 *(A), *ALP *(B), *OPN *(C) and *COL1A1 *(D) in mesenchymal stem cells (MSCs) cultured in osteogenic differentiation medium with 0.5 μM naproxen (Npx) for 6 and 12 days.** The results are shown as mean ± SD of three independent experiments with MSCs from three different donors. *P <0.05; **P <0.01 cells cultured with both cyclopamine (Cpn) and naproxen (Npx) versus cells cultured with Npx only.

Cpn can downregulate HH signaling pathway activity by binding directly to SMO, a key component of the HH signaling pathway [27,28]. To determine whether the effect of Npx on the expression of *COL10A1*, *ALP*, *OPN* and *COL1A1* was directly through the HH signaling pathway, MSCs were cultured in osteogenic differentiation medium with Npx alone or both Npx and Cpn. On day 6, the expression of *COL10A1* with cyplocamine supplementation decreased significantly compared with Npx alone (*P* = 0.04) (Figure 5A). *ALP* expression was increased significantly with Cpn supplementation compared with Npx alone on day 6 (*P* = 0.003) and day 12 (*P* = 0.002) (Figure 5B). Compared with Npx alone, the expression of *OPN* was decreased significantly by Cpn on both day 6 (*P* <0. 001) and day 12 (*P* = 0.007) (Figure 5C). The expression of *COL1A1* was upregulated by Cpn on day 6 (*P* = 0.002) and day 12 (*P* = 0.04). The significant difference was not observed for either *COL10A1* (Figure 5A) or *ALP* (Figure 5B) expression with Cpn supplementation on day 12. Thus Npx appeared to affect the expression of *COL10A1*, *ALP*, *OPN* and *COL1A1* through HH signaling in MSCs. Preliminary studies showed that Cpn alone had no effect on *COL10A1*, *ALP*, *OPN* and *COL1A1* expression (data not shown), and because the results of culturing MSCs in osteogenic differentiation medium alone were conducted in Figure 1, these were not included in order to simplify the figures.

## Discussion

If MSCs are to be used to stimulate bone repair in the presence of NSAIDs in the synovial fluid, it is essential that NSAIDs do not interfere with osteogenic differentiation. The results of the present work indicate that Npx can induce the expression of *COL10A1* and *OPN*, and downregulate the expression of *ALP* and *COL1A1* through activation of HH signaling. The downregulation of *ALP* gene expression led to the suppression of its activity and decreased mineral deposition. Osteogenesis occurs through an endochondral process involving cellular hypertrophy and mineralization in a manner analogous to the growth plate. Type X collagen is a marker of hypertrophy, whereas ALP, OC, OPN and COL1A1 are bone matrix proteins synthesized by osteoblasts [24-26]. Mineralization is a functional endpoint reflecting advanced osteogenesis. Achieving increased expression of OC, OPN, COL1A1, as well as ALP activity and mineralization is therefore an essential requisite for bone repair. Thus Npx has the potential to interfere with MSCs in repairing bone.

The effect of anti-inflammatory drugs on the osteogenesis of MSCs has been reported previously in other studies. It was reported that the osteogenic potential of MSC is inhibited/delayed by treatment with high-dose NSAIDs under inflammatory conditions, while no significant effects were observed in non-inflammatory-conditioned MSCs [29]. In another experiment, it was reported that NSAIDs can inhibit bone formation via blockage of MSC chondrogenic differentiation - an important intermediate phase in normal endochondral bone formation - but not the osteogenesis of MSC [30]. In our study, Npx had a tendency to downregulate matrix mineralization. Furthermore, its effect on the expression of *OPN* and *COL1A1* could affect the function of bone. In addition to their effects on osteogenesis, high-dose and long-term administration of NSAIDs has been shown to induce adipogenesis in stem cells. For instance, Indomethacin can stimulate adipogenesis of MSCs [31]. However, the effect of Npx on adipogenesis is not known. Interestingly, Npx was shown to have anti-obesity effects in animals when injected at a high dose [32].

A decrease in the expression of osteogenic markers and functional mineralization, and an increase in the expression of hypertrophic chondrocyte markers was observed after prolonged culture with Npx. During hypertrophy, cells enlarge in many tissues such as the growth plate, muscle and cartilage, which also occurs in conditions of degeneration, such as OA. However, we did not observe any appreciable increase in cell volume. It has been reported that calcium/calmodulin-dependent kinase II (CamkII) is an essential component of intracellular signaling pathways regulating chondrocyte maturation, and it can induce cell hypertrophy through a branched set of effector pathways including the transcriptional regulators Runx2 and Mef2c [33,34]. Activation of CamkII activity can result in premature hypertrophic gene expression, with no cell swelling. Furthermore, chondrocytes can display morphological changes consistent with hypertrophy, without upregulating the expression of hypertrophic genes [33,34]. Thus, the hypertrophic program may be more complex than previously thought.

Npx affected the expression of *IHH*, *PTC1*, *GLI1* and *GLI2* genes belonging to the HH signaling pathway, and affected the expression of osteogenic genes in MSCs at different time points. This might be the result of the interaction of different proteins involved in HH signaling [22]. The upregulation of *IHH*, *GLI1* and *GLI2* can increase the HH signaling pathway positively, while the upregulation of *PTC1* has a negative effect on the HH signaling function as more IHH molecules will interact with increased PTC1 and result in less SMO being released. As Npx did not affect the expression of *GLI3*, it is possible that GLI3 does not play a direct role in the osteogenesis of MSCs. Interestingly, we were unable to detect the expression of Sonic Hedgehog (*SHH*), another gene involved in HH signaling and osteogenesis of MSCs, with primers that were specific for human *SHH*.

The bone repair process in adults resembles normal development of the skeleton during embryogenesis. Numerous signaling pathways induce the osteogenic differentiation of MSCs. Although the mechanisms have not been fully discerned, and only HH signaling was studied in this research, other signaling pathways may also affect stem cell differentiation. As the results in this study indicate that Npx can affect treatment and lineage differentiation of MSC through the IHH signaling pathway, a better understanding of these underlying mechanisms and the effect of other NSAIDs on the osteogenesis of MSCs have far-reaching implications for improving bone repair for OA treatment.

## Conclusions

The results support the concept that Npx has a dual role in that it can stimulate hypertrophic differentiation of MSCs, while suppressing osteogenic differentiation of MSCs and mineral deposition in the matrix through HH signaling.

## Abbreviations

ALP: alkaline phosphatase; bp: base pairs; COL1A1: collagen I, type 1; COL10A1: collagen X, type 1; Cpn: cyclopamine; DMEM: Dulbecco’s modified Eagle’s medium; FBS: fetal bovine serum; GAPDH: glyceraldehyde-3-phosphate dehydrogenase; GLI: GLI-Kruppel family member GLI; IHH: Indian hedgehog; MSC: mesenchymal stem cell; Npx: naproxen; NSAIDs: non-steroidal anti-inflammatory drugs; OA: osteoarthritis; OC: osteocalcin; OPN: osteopontin; PBS: phosphate-buffered saline; PCR: polymerase chain reaction; PTC1: Patched-1; SMO: Smoothened.

## Competing interests

The authors declare that they have no competing interests.

## Authors’ contributions

OS cultured the cells, performed the real-time RT-PCR, and analyzed the ALP activity. HTW designed the experiments, analyzed the data, and wrote the manuscript. AMA cultured the cells, and performed the real-time RT-PCR. IH and OC performed the real-time RT-PCR experiments and statistical analysis. RG cultured the cells and analyzed enzyme activity. JA designed the experiment, and revised the manuscript. FM conceived and supervised the whole study and finished writing the manuscript. All authors read and approved the final manuscript.
